# Emotion regulation and moral and social rule awareness in early childhood: an exploratory correlational study

**DOI:** 10.3389/fpsyg.2026.1792202

**Published:** 2026-04-10

**Authors:** Gökhan Güneş, Ali Ammar Kurt, Buket Özkan

**Affiliations:** 1 Faculty of Education, Department of Early Childhood Education, Mersin University, Mersin, Türkiye; 2 Faculty of Education, Department of Guidance and Psychological Counseling, Mersin University, Mersin, Türkiye

**Keywords:** early childhood development, emotion regulation, moral and social rule conception, preschool education, socio-emotional competence

## Abstract

Early childhood represents a critical developmental period during which children begin to internalize social norms, understand moral principles, and acquire emotion regulation skills that guide adaptive behavior. This exploratory correlational study examined the associations between preschoolers’ emotion regulation skills and their perceptions of moral and social rules. The participants were 45 children (21 girls, 24 boys; *M* = 67.47 months) attending two public preschools. Data were collected using the Moral and Social Rule Perception Scale and the Emotion Regulation Scale. Results indicated that children displayed high levels of moral and social rule conception and moderate levels of emotion regulation. Pearson correlation analyses revealed significant positive associations between emotion regulation skills-particularly the use of emotion regulation strategies-and both moral and social rule conceptions. The strongest correlation was observed between emotion regulation strategies and total moral and social rule conception (*r* = 0.78, *p* < 0.001), suggesting that strategic emotional competence is closely linked to children’s normative awareness. These exploratory findings indicate that emotional competence-particularly strategy use-may be meaningfully associated with children’s moral and social rule conception during early childhood. However, given the small sample size (*N* = 45), the cross-sectional design, and the exploratory nature of the analyses, causal or predictive claims cannot be made. Limitations include the context-specific sample and reliance on a single assessment occasion. The study provides preliminary, hypothesis-generating insights for educators, counselors, and child service practitioners, emphasizing the potential importance of integrating socio-emotional learning components into early education and child development programs to support adaptive moral and social functioning.

## 1 Introduction

Early childhood represents a pivotal developmental stage during which individuals simultaneously cultivate their moral judgments, internalize social norms, and develop emotion regulation skills (Schiele et al., 2024). In this period, while all developmental domains evolve rapidly, the foundations of emotional, moral, social, and normative development are also established (Schiele et al., 2025). Importantly, moral and social rule conception during this period should be understood as emerging and actively developing capacities rather than fixed or fully formed traits; children at this age are in the process of transitioning from externally regulated to increasingly self-regulated moral and emotional agents (Eisenberg et al., 2010; Kochanska and Knaack, 2003). Children’s perception of moral rules refers to their capacity to recognize norms grounded in justice, fairness, and nonmaleficence, as well as to regulate their behaviors in accordance with these principles. In contrast, social rule perception encompasses the understanding of authority- and context-dependent norms, such as social conventions, routines, and etiquette (Langenhoff et al., 2022; Zhang et al., 2023). The fundamental distinction between moral and social rules provides a critical framework for understanding normative cognitive development in early childhood. While moral rules are typically based on principles of fairness and the avoidance of harm to others, social rules are contingent upon authority and situational context. Learning to differentiate between these two types of norms is essential for children’s behavioral self-regulation and for the organization of social relationships (Takagi and Saltzstein, 2021; Tomasello, 2025). The literature underscores the importance of supporting the internalization of moral behaviors such as honesty, refraining from deception, and adhering to established rules (Asendorpf and Nunner-Winkler, 1992; Augustine and Stifter, 2015; Dong et al., 2021; Wang et al., 2022). In addition to these, relevant studies emphasize that prosocial behaviors may also reflect moral behavioral patterns. Acts such as sharing or offering help are conceptualized as both moral and prosocial behavioral schemas (Reis and Sampaio, 2019; Tan et al., 2020; Wildeboer et al., 2017). Sharing, for instance, is regarded as a behavioral manifestation associated with moral reasoning and illustrates how young children can enact and evaluate normative concepts of fairness. Moreover, several studies have pointed out that antisocial behaviors may reflect the opposite-immoral behavioral patterns-highlighting the developmental continuum between prosocial and antisocial expressions of morality (Augustine and Stifter, 2015; Colasante et al., 2015; Stifter et al., 2009).

During early childhood education, children function as moral agents, actively engaging in behaviors that reflect emerging ethical understanding. This developmental stage supports the growth of fundamental social competencies such as social adjustment, empathy, and self-regulation, which, in turn, have direct implications for individuals’ social functioning in later life (Howard et al., 2024; Korucu et al., 2022; Simon and Nader-Grosbois, 2021). Therefore, the interaction between children’s individual emotional and cognitive capacities and the quality of their educational environment should be regarded as a critical factor associated with their ability to comprehend and act in accordance with moral and social rules. A lack of stability and order within preschool environments often manifests in difficulties adhering to rules, leading to challenges in classroom management as well as social issues such as peer conflicts and bullying (Cutler et al., 2022; Devlin et al., 2024; Kim et al., 2020; McGoey et al., 2023; Wulan and Fridani, 2021). Such instability negatively influences both children’s individual development and their processes of socialization (Kim et al., 2020; McGoey et al., 2023). In this context, it becomes crucial for children to understand moral and social rules and to regulate their emotions and behaviors appropriately when confronted with moral dilemmas or conflicts (Cai and Meng, 2024; Liman, 2024). The dynamic interplay between children’s personal characteristics and emotional competencies and the classroom climate and social environment they experience stands as one of the core factors associated with their understanding of and adherence to moral and social norms. Clear and consistent rules, supportive teacher-child relationships, and positive peer interactions facilitate the use of emotion regulation skills and the internalization of moral and social norms. Conversely, ambiguous rules and insufficient emotional support may increase the risk of dysregulation, resulting in more frequent classroom conflicts and antisocial behaviors (Bailey et al., 2022; Hosokawa et al., 2024; Liman, 2024; Somerville et al., 2024).

From a theoretical perspective, Eisenberg et al. (2000) emphasized that the ability to regulate emotions is closely associated with moral development and moral behavior. In this context, the concept of self-regulation, which encompasses emotional regulation, refers to the capacity to control one’s thoughts, emotions, and behaviors. Accordingly, children with strong self-regulation skills are more capable of managing their emotions and, indirectly, of shaping their perceptions of moral and social rules. They may also demonstrate the ability to act morally even when such behavior conflicts with their self-interest, suggesting a relationship between rule internalization, compliance, and self-regulatory capacity (Kochanska and Knaack, 2003; Schütz and Koglin, 2023). These skills develop during early childhood, increase rapidly throughout the preschool years, and continue to evolve in complex ways into adulthood (Denham et al., 2014; Nigg, 2017). To understand the link between self-control and moral behavior, it is essential to examine how children recognize, express, and manage their emotions. Emotion regulation encompasses the ability to identify, label, express, and appropriately manage emotional experiences (Acar and Arslan, 2022). These abilities allow children to behave adaptively in social interactions and to demonstrate self-control in stressful or conflictual situations. The subcomponents of emotion regulation include (a) recognition and understanding of emotions, (b) emotional expression, and (c) use of appropriate regulation strategies. Recognition and understanding enable children to comprehend both their internal emotional states and the emotional signals of others; expression involves the socially appropriate communication of these emotions; and regulation strategies entail producing context-appropriate responses and managing negative emotions (Aghaziarati and Nejatifar, 2023; Nadia and Popivanov, 2025; Richard et al., 2025). These subcomponents play distinct yet interconnected roles in children’s adaptation to social norms and emotional adjustment. Scholars have consistently identified emotion regulation as a core developmental factor essential for maturation (Cole et al., 2004) and as closely linked to the development of social competence, academic success, and psychosocial adjustment (Eisenberg et al., 2010). Emotion regulation strategies are also vital indicators of mental health across both child and adult populations (Aldao et al., 2010; Bender et al., 2012; Espenes et al., 2025). Children with strong emotion regulation skills manage their emotions more effectively and consequently display more prosocial, cooperative, and rule-abiding behaviors. A study investigating both direct and empathy-mediated effects of emotion regulation revealed that these skills are directly associated with children’s prosocial behaviors (Vecchio et al., 2023). Prosocial behaviors-such as sharing, helping, comforting, and protecting-are characterized by a willingness to benefit others (Eisenberg et al., 2015). Children who effectively employ these skills demonstrate awareness of both their own and others’ emotions and understand how to express them appropriately within context (Gross, 2013). Conversely, children who fail to regulate their emotions are more likely to exhibit impulsivity, aggression, peer bullying, classroom disruptions, and, consequently, social rejection (Ersan and Tok, 2019; Salerni and Messetti, 2025; Qashmer, 2023). Emotional and behavioral problems associated with social rejection are closely linked to difficulties in emotion regulation. In this sense, emotion regulation serves a functional role by enabling children to respond flexibly to environmental stimuli and to display behaviors aligned with social norms (Cole et al., 1994). This ability contributes to peer acceptance and the improvement of interpersonal relationships (Blair et al., 2015; Blandon et al., 2010; Kim and Cicchetti, 2010). In other words, the capacity to regulate one’s internal states is associated with the selection and execution of contextually appropriate behaviors while supporting peer acceptance (Eisenberg et al., 2007; Rose-Krasnor and Denham, 2009). Empirical findings have shown that emotion regulation plays a significant role in peer acceptance-a key component of social and emotional development (Coie et al., 2006; Kim and Cicchetti, 2010; Liang et al., 2025). Emotion regulation strategies reflect emotional competence and function as a primary correlate of the quality of peer relationships (Gülay Ogelman and Fetihi, 2021). The emotional social competence model supports this view, emphasizing that emotion regulation strategies in early childhood serve a facilitative role in peer dynamics. Moreover, the ability to regulate emotions functionally from early childhood onward contributes to social adaptability and sustained peer acceptance in adulthood (Izard, 2001). Conversely, deficits in emotional regulation are associated with behavioral problems and difficulties in social relationships (Granic et al., 2012; Lengua, 2003). Robust evidence supports the association between externalizing behavioral problems and inadequate emotion regulation (Halligan et al., 2013; Mihic and Novak, 2018). In their seminal longitudinal study, Trentacosta and Shaw (2009) examined emotion regulation as a key variable in the emergence of antisocial behaviors and social rejection. Their model, tested from early childhood through adolescence, employed empirical tasks assessing regulatory skills and a sociometric technique (“peer nomination”) to measure social rejection, incorporating reports from parents and teachers. The results revealed that social rejection was directly related to emotion regulation capacity and that this variable exerted indirect effects on behavioral difficulties through its mediating role. These findings collectively suggest that emotion regulation is closely associated with behavioral adjustment and may be a critical factor in the cognitive perception of moral and social rules. Furthermore, externalizing problems linked to social rejection-mediated by poor emotional regulation-may undermine children’s ability to act according to rules and make moral judgments in conflict situations. In this light, children with stronger emotion regulation skills may be better positioned to internalize social norms more effectively and display greater resistance to antisocial behavior. In this framework, the relationship between children’s emotion regulation skills and their perceptions of moral and social rules emerges as a critical area of investigation for understanding children’s behaviors and developing effective support strategies. In their meta-analysis, Schütz and Koglin (2023) observed that studies exploring the connection between emotion regulation and morality remain limited and heterogeneous, indicating a significant gap in the literature. Prior research has shown a small but positive relationship between self-regulation and morality, with sub-analyses involving emotion regulation revealing the strongest links to moral cognition and behavior. The ability to regulate emotions also facilitates the development of empathy and sympathy, enhancing the capacity to understand others’ perspectives and recognize their needs-key processes that positively influence moral reasoning. Conversely, insufficient emotion regulation may lead to excessive empathic arousal, resulting in internal distress and shifting cognitive focus from moral action to self-soothing, thereby negatively impacting moral cognition and behavior. Overall, these findings indicate that well-developed emotion regulation skills can support moral behavior in the context of moral conflict, underscoring the importance of fostering effective emotion regulation strategies. Accordingly, the present study aims to explore the associations between children’s emotion regulation skills and their perceptions of moral and social rules. In line with this aim, the research questions were formulated as follows:

1. Is there a significant relationship between children’s emotion regulation skills and their perceptions of moral and social rules?
2. What is the strength and direction of the associations between specific emotion regulation subdimensions and children’s moral and social rule conceptions?

Through this study, it is intended to make a meaningful scientific contribution to the literature by exploring the associations between children’s emotional and cognitive regulation processes and the internalization of moral and social norms.

### 1.1 Purpose and significance of the study

The primary aim of this study is to examine the relationship between moral and social rule perception and emotion regulation skills during early childhood. Through findings obtained on the link between preschool children’s perceptions of moral and social rules and their emotion regulation skills, this study offers valuable insights into the function of emotional regulation in the process of adapting to moral and social norms. The results of this research provide a holistic framework for understanding children’s socio-emotional development and hold the potential to inform future theoretical models and educational programs. Early childhood represents a critical developmental period during which children intensely learn and internalize social norms, offering important opportunities for the acquisition of foundational skills necessary for both individual and social adaptation. The significance of this study can be articulated in three dimensions. First, examining the relationship between emotion regulation skills and moral and social rule perception provides essential data for understanding how normative awareness and emotional competence co-develop in children aged 5–6 years. Second, the findings contribute to strengthening the theoretical framework explaining the association between children’s emotion regulation skills and their perceptions of moral and social rules. This allows for a more detailed exposition of the role of emotional regulation in normative cognition and supports a more detailed understanding of early childhood normative development processes more clearly in relation to the subdimensions of emotion regulation-namely, recognition and understanding of emotions, emotional expression, and regulation strategies. Finally, the data obtained from this study may serve as a theoretical foundation for future educational interventions and supportive programs. For instance, activities designed to enhance emotion regulation skills may strengthen children’s capacity to comprehend moral and social rules and foster their normative awareness. It should be noted, however, that this study does not include direct intervention or classroom observation components.

## 2 Method

### 2.1 Sample

The sample of this research was determined using the purposeful sampling method. Purposeful sampling is a non-probabilistic technique that enables the selection of participants possessing specific qualitative characteristics deemed most suitable for the research purpose (Baltacı, 2019). As the primary aim of the study was to examine the relationship between preschool children’s emotion regulation skills and their perceptions of moral and social rules, institutions that allowed direct access to children within this age group and were suitable for observational data collection were preferred. Accordingly, the sample consisted of 45 children attending two different public preschools. Among the participants, 24 were boys and 21 were girls, with ages ranging from 64 to 72 months (*M* = 67.47 months). This age range represents a critical developmental period in which children acquire key competencies in both socio-emotional and moral development (Hosokawa et al., 2024). The following criteria were considered in the selection of participants:

- Active attendance at a preschool institution
- Belonging to a mid-level developmental risk group (i.e., without diagnosed developmental delays or advanced conditions)
- Possessing sufficient developmental capacity to comprehend data collection tools (e.g., scale applications, observational tasks)
- Obtaining written parental consent
- Participation of institutions that granted permission and demonstrated willingness to collaborate.

In addition to these inclusion criteria, practical considerations such as accessibility, time management, institutional cooperation, and sustainability of the data collection process were also taken into account. Consequently, the sample was structured to include children capable of providing qualified data while ensuring logistical feasibility for the research process. This approach not only enhanced the content validity of the study but also facilitated the execution of a field-compatible and ethically sound research process. Written informed consent was obtained from the parents of all participating children, and ethical approval for the study was granted by the Ethics Committee of the researchers’ affiliated university (Approval No. 133).

### 2.2 Research design and data collection instruments

This study was conducted using the relational survey model, one of the quantitative research methods (Creswell and Creswell, 2018; Garip, 2023). As noted by Fraenkel and Wallen (2009), relational or correlational research allows investigators to examine relationships among variables without attempting to influence them, providing a clear statistical picture of how closely changes in one variable are associated with changes in another within a specific sample. Two instruments were used for data collection: the Moral and Social Rule Conception Scale, originally developed by Smetana (1985) and adapted to Turkish culture by Seçer et al. (2006), and the Emotion Regulation Scale, developed by Acar and Arslan (2022). The Moral and Social Rule Conception Scale consists of a total of 10 items, including two subscales: the Moral Rule Conception subscale (5 items) and the Social Rule Conception subscale (5 items). Within the scope of this study, Cronbach’s alpha coefficients for this scale were calculated as 0.82 for the Moral Rule Conception subscale, 0.87 for the Social Rule Conception subscale, and 0.91 for the overall scale. The Emotion Regulation Scale includes three subdimensions: Recognition and Understanding of Emotions, Labeling (Expression) of Emotions, and Emotion Regulation Strategies. Each of these subdimensions assesses six emotions-sadness, fear, anger, disappointment, anxiety, and shyness-through scenario-based items. In the Recognition and Understanding and Labeling (Expression) subscales, each emotion is measured through a single scenario (six items per subscale). In the Emotion Regulation Strategies subscale, three different scenarios are presented for each of the six emotions (a total of 18 scenarios), each scored according to four positive and four negative response options. The Cronbach’s alpha coefficient of the Emotion Regulation Scale was calculated as 0.80. Internal consistency coefficients above 0.70 indicate that the instruments used in the study possess sufficient reliability for research purposes (Cheung et al., 2024).

### 2.3 Data analysis

Since the skewness coefficient values of all total and subdimension scores from the two instruments administered in the study were within the range of ±2.0, and the kurtosis coefficient values were within ±7.0, the data set was considered to meet the assumptions of normal distribution (Byrne, 2016; Hair et al., 2010; Kline, 2011). Accordingly, analyses addressing the research questions were conducted using parametric tests, as the data satisfied normality assumptions. Given the exploratory nature of this study and the relatively small sample size (*N* = 45), the primary analytical strategy was based on Pearson product–moment correlation coefficients to examine the strength and direction of bivariate associations between variables. This correlational approach was deemed the most appropriate analytical framework for the current sample, as it avoids the risks of model overfitting, parameter instability, and inflated Type I error rates associated with more complex inferential procedures (e.g., multiple regression, ANOVA) when applied to small samples. Effect sizes were interpreted using Cohen (1988) guidelines, whereby *r* = 0.10 indicates a small effect, *r* = 0.30 a medium effect, and *r* = 0.50 a large effect. All analyses were conducted using SPSS (Version 25), and the significance level was set at *p* < 0.05.

## 3 Findings and discussion

Descriptive statistical values for the Moral and Social Rule Conception Scale are presented in Table 1.

**Table 1:** Descriptive statistics for the moral and social rule conception scale.

Moral and social rule conception scale	Range	Min.	Max.	$\bar{x}$	SD	S	Skew.	Kurt.	%
Conceptions of moral rules	17.00	23.00	40.00	36.04	4.43	19.59	−1.37	1.48	76.73
Conception of social rules	35.00	5.00	40.00	30.91	6.72	45.17	−1.49	3.90	74.03
Total of conception of moral and social rules	49.00	31.00	80.00	66.96	10.23	104.73	−1.34	2.25	73.38

When the results of the Moral and Social Rule Conception Scale administered to 45 preschool children were examined, it was found that children demonstrated a high level of moral rule conception, with an average score of 36.04 (SD = 4.43), corresponding to 76.7% of the maximum possible score. The relatively low standard deviation (SD = 4.43) indicates a fairly consistent distribution of moral rule conception among participants. In contrast, children’s social rule conception scores averaged 30.91 (SD = 6.72), representing 74.0% of the maximum possible score. However, the higher standard deviation (SD = 6.72) suggests greater individual variability in social rule conception compared to moral rule conception. For the total scale score, the mean was 66.96 (SD = 10.23), corresponding to a 73.4% overall rule conception level. The relatively higher standard deviation for the total score (SD = 10.23) indicates more pronounced differences among children in their conceptions of moral and social rules. Taken together, these descriptive results indicate relatively high moral and social rule conception scores in this sample of preschool children, although individual differences appear to be more pronounced in social rule conception.

In the study, it was observed that preschool children generally exhibited high levels of moral and social rule conception, with moral rule conception showing a more consistent distribution than social rule conception. This finding suggests that during early childhood, children demonstrate relatively more stable development in understanding and internalizing moral norms (Gniewosz et al., 2023).

The literature highlights that moral development in this period is critical for learning fundamental social norms and transforming them into personal values (Yalcin, 2021). The greater variance observed in social rule conception indicates differences among children in how they interpret and internalize social rules. This variation can be attributed to the context-dependent and experience-sensitive nature of social rules (Langenhoff et al., 2022). Furthermore, the consistency in moral rule conception has been linked to children’s understanding of justice and rights within social interactions (Killen and Dahl, 2021). In contrast, social rules primarily serve to maintain social order and promote conformity, which may lead children to develop different perceptions depending on environmental influences (Collyer et al., 2022). In this respect, the findings of the present study align with previous research, indicating that preschool children display positive conceptions of both moral and social rules, yet social rule conception tends to vary more strongly across individuals due to personal and contextual factors (Lee, 2023; Smetana, 1985, 2006).

Descriptive statistics for the Emotion Regulation Scale are presented in Table 2.

**Table 2:** Descriptive statistics for the emotion regulation skills scale.

Emotion regulation skills scale	Range	Min.	Max.	$\bar{x}$	SD	S	Skew.	Kurt.	%
Recognizing and understanding emotions sub-dimension	9.00	6.00	15.00	10.64	2.28	5.19	−0.31	−0.45	51.60
Expressing emotions sub-dimension	9.00	6.00	15.00	9.22	1.91	3.63	0.72	1.41	35.80
Emotion regulation strategies sub-dimension	70.00	67.00	137.00	96.64	17.81	317.14	0.40	−0.12	42.35
Total of emotion regulation skills scale	80.00	84.00	164.00	116.51	19.78	391.34	0.48	0.13	40.64

In the study, the mean score of children on the Recognizing and Understanding Emotions subdimension was 10.64 (SD = 2.28), indicating a moderate level of development (51.60%) with relatively consistent scores among participants. This suggests that children’s skills in this domain were generally uniform across the group. For the Expressing Emotions subdimension, the mean score was 9.22 (SD = 1.91), corresponding to a low performance level (35.80%) with similarly close score distributions, indicating limited variability among children. In the Emotion Regulation Strategies subdimension, the mean score was 96.64 (SD = 17.81), reflecting a low developmental level (42.35%) and higher standard deviation values, suggesting greater individual differences in this domain. The total emotion regulation skills score was 116.51 (SD = 19.78), corresponding to a low overall level (40.64%) and showing considerable variability among children. These results indicate that preschool children demonstrate moderate development in recognizing and understanding emotions but lower levels of development in expressing emotions and using emotion regulation strategies. Notably, individual differences are most pronounced in the area of strategy use, highlighting this as a potential focus for early emotional support and intervention.

The findings revealed that preschool children demonstrated moderate development in recognizing and understanding emotions, but low levels of development in expressing emotions and employing emotion regulation strategies. These results are consistent with the findings of researchers such as Richard et al. (2025), who reported that children tend to acquire adequate skills in recognizing and understanding basic emotions during early childhood. However, the lower performance observed in the Expressing Emotions and Emotion Regulation Strategies subdimensions may be attributed to the greater complexity and gradual developmental nature of these skills (Kao et al., 2024). Moreover, the substantial individual differences identified in the use of emotion regulation strategies suggest diversity in children’s social, cognitive, and emotional experiences (Oğuz and Pınar, 2025). In this context, supporting and fostering emotion regulation skills during early childhood is essential for promoting better socio-emotional adjustment and adaptive development.

To examine the associations between children’s moral and social rule conceptions and their emotion regulation skills, Pearson correlation analyses were conducted. The results are presented in Table 3.

**Table 3:** Pearson correlation analysis results for the relationship between children’s moral and social rule conceptions and emotion regulation skill.

		Emotion regulation skills scale			
	Subscale	Recognizing and understanding emotions sub-dimension	Expressing emotions sub-dimension	Emotion regulation strategies sub-dimension	Total of emotion regulation skills scale
Moral and social Rule Conception Scale	Conceptions of moral rules	0.407^**^	0.228	0.687^**^	0.688^**^
	Conception of social rules	0.427^**^	0.364^*^	0.737^**^	0.747^**^
	Total of moral and social rule conception scale	0.457^**^	0.337^*^	0.781^**^	0.788^**^

^**^p<0.01.

According to Table 3, a moderate positive and significant correlation was found between the Moral Rule Conception subdimension and the Recognizing and Understanding Emotions subdimension (*r* = 0.407, *p* < 0.001), while strong positive and significant correlations were observed with the Emotion Regulation Strategies subdimension (*r* = 0.687, *p* < 0.001) and the total emotion regulation score (*r* = 0.688, *p* < 0.001). In contrast, the correlation between Moral Rule Conception and the Expressing Emotions subdimension was weak and not statistically significant (*r* = 0.228, *p* > 0.05). The Social Rule Conception subdimension showed a moderate positive correlation with Recognizing and Understanding Emotions (*r* = 0.427, *p* < 0.001); a low-to-moderate positive correlation with Expressing Emotions (*r* = 0.364, *p* < 0.05); and strong positive and significant correlations with Emotion Regulation Strategies (*r* = 0.737, *p* < 0.001) and the total emotion regulation score (*r* = 0.747, *p* < 0.001). Similarly, the total score of the Moral and Social Rule Conception Scale exhibited consistent patterns of association with emotion regulation skills: a moderate correlation with Recognizing and Understanding Emotions (*r* = 0.457, *p* < 0.001); a low-to-moderate correlation with Expressing Emotions (*r* = 0.337, *p* < 0.05); and strong positive correlations with both Emotion Regulation Strategies (*r* = 0.781, *p* < 0.001) and the total emotion regulation score (*r* = 0.788, *p* < 0.001).

The high correlation (*r* = 0.78) identified between emotion regulation and moral/social rule awareness likely reflects a fundamental developmental integration between these two domains during the preschool years. Theoretically, emotion regulation may function as a foundational self-regulatory capacity, associated with children’s ability to manage impulsive tendencies and allocate cognitive resources toward the comprehension of social expectations and moral mandates (Eisenberg et al., 2015; Killen and Smetana, 2015). This finding aligns with integrative-developmental frameworks, which conceptualize emotional competence and social cognition as interdependent systems. Specifically, as children’s regulatory capacities mature, they gain the psychological distance and cognitive clarity required to evaluate the underlying logic of moral norms. This cognitive shift may support a gradual transition from superficial obedience to a more authentic internalization of social values (Thompson, 2014). Consequently, our results suggest that emotion regulation may function not merely as a proxy for rule awareness, but as a developmental capacity closely linked to moral understanding. Nevertheless, we acknowledge that the potential for partial measurement overlap between evaluative instruments cannot be entirely discounted; thus, future research utilizing multi-method designs is essential to further isolate genuine developmental associations from potential measurement artifacts.

These findings reveal a particularly strong relationship between emotion regulation strategies and both moral and social rule conceptions, suggesting that emotion regulation skills may be closely linked to children’s adaptation to social norms and moral reasoning processes. The results indicate that preschool children’s moral and social rule conceptions are positively and significantly associated with their emotion regulation skills. In particular, the strong correlations between Emotion Regulation Strategies and both Moral Rule Conception (*r* = 0.687) and Social Rule Conception (*r* = 0.737) suggest that emotional processes are closely associated with children’s adherence to social norms. This finding supports previous studies demonstrating that emotion regulation skills are closely linked to social competence and rule-consistent behavior (Martinsone et al., 2022; Oğuz and Pınar, 2025; Salerni and Messetti, 2025). The moderate correlation between Moral Rule Conception and Recognizing and Understanding Emotions (*r* = 0.407) further indicates that children’s moral evaluations rely not only on cognitive processes but also on emotional awareness mechanisms. Consistent with this, Schütz and Koglin (2023), Gungordu et al. (2025), and Malti and Speidel (2024) emphasized that children’s empathic awareness and emotional self-regulation skills are closely associated with the development of internalized morality. In this sense, recognizing and understanding emotions may be an important correlate of children’s ability to select appropriate and socially accepted behaviors in interpersonal contexts. The significant correlations identified between Social Rule Conception and all subdimensions of the Emotion Regulation Skills Scale suggest that the enactment of social rules is closely tied to emotion regulation.

In preschool contexts, behaviors such as maintaining positive peer relationships, participating in group interactions, and complying with teacher guidance require the ability to control emotions and generate contextually appropriate responses (Herndon et al., 2013; Nilfyr and Ewe, 2025; Wilhelmsen et al., 2025; Xu and Qiao, 2025). This indicates that social rule adherence depends not only on external behavioral compliance but also on internal emotional management. The weak-to-moderate correlations between Expressing Emotions and rule conception may suggest that the development of emotional expression lags behind the internalization of cognitive and social norms or that this ability is more sensitive to individual traits than to social interaction. Supporting this interpretation, Herndon et al. (2013), Silkenbeumer et al. (2024), Wu and Gweon (2021), and Chaplin et al. (2017) have emphasized that developmental differences in children’s emotional expression are pronounced and shaped largely by social context. Overall, the present study suggests that the developmental sophistication of emotion regulation skills-particularly at the strategic level-may serve as a key correlate of children’s adaptation to moral and social norms. The descriptive and correlational findings collectively underscore that the processes underlying moral and social rule compliance in early childhood are grounded not only in behavioral but also in emotional foundations. Accordingly, future educational programs and theoretical models should place socio-emotional competencies at the center of moral and social development frameworks.

### 3.1 Contextual discussion

The findings of the present study should be interpreted within the broader developmental and cross-cultural context of research on moral cognition and emotion regulation. The strong positive associations observed between emotion regulation strategies and both moral and social rule conceptions are consistent with findings from diverse cultural settings. Research conducted in Western European (Schiele et al., 2024, 2025; Diebold et al., 2025), East Asian (Dong et al., 2021; Zhu et al., 2021), and Middle Eastern (Qashmer, 2023) contexts has similarly highlighted the centrality of emotion regulation in children’s social and moral development. The present study, conducted with Turkish preschoolers, extends this evidence to a relatively underrepresented cultural context in the developmental literature.

However, the cultural specificity of moral and social rule distinctions warrants careful consideration. Smetana (1985, 2006) social-cognitive domain theory, which underpins the measurement framework used in this study, was originally developed and validated primarily in Western cultural contexts. Although the Turkish adaptation by Seçer et al. (2006) demonstrated adequate psychometric properties, the degree to which the moral-social rule distinction maps onto Turkish cultural norms and child-rearing practices remains an important question for future research. Cultural values regarding authority, collectivism, and social harmony may shape how children perceive and internalise different types of rules (Langenhoff et al., 2022; Tomasello, 2025).

The developmental period examined in this study (64–72 months) represents a particularly sensitive window for the co-development of emotion regulation and normative understanding. During this period, children are transitioning from externally regulated to increasingly self-regulated emotional and moral agents (Eisenberg et al., 2010; Kochanska and Knaack, 2003). The pattern of correlations observed here with emotion regulation strategies showing stronger associations than emotion recognition or expression is consistent with the theoretical proposition that it is the active, strategic management of emotions, rather than mere awareness, that facilitates the internalisation of moral and social norms (Schütz and Koglin, 2023). This finding resonates with Eisenberg et al. (2000) model, which emphasises that the functional deployment of regulatory strategies, rather than the passive experience of emotional states, is what supports adaptive moral behavior.

Smetana’s distinction between moral and social rules provides a valuable framework; however, when considering the Turkish cultural context, it is necessary to account for specific relational dynamics. In Türkiye, children grow up within strong relational networks in which respect for elders, attentiveness to others’ feelings, and a sense of responsibility toward close social circles are not merely matters of etiquette but internalized obligations. Consequently, for a Turkish child, neglecting to greet an elder or failing to meet a social expectation may be experienced not as the violation of an arbitrary convention but as causing harm to a valued relationship. This indicates that in cultural settings where interpersonal connection and social harmony are central, the boundary between social conventions and moral principles may become less clearly differentiated. What Smetana might categorize as a social convention can, in such contexts, be perceived by children as a moral imperative, insofar as maintaining relationships is regarded as a moral responsibility.

## 4 Conclusion

This study examined associations between preschool children’s emotion regulation skills and their moral and social rule conception. Overall, higher emotion regulation scores-particularly strategy use-were associated with higher moral and social rule conception scores in this sample; however, the design does not allow causal conclusions.

Descriptive analyses indicated that children in this sample demonstrated relatively high levels of moral rule conception (76.73%) and social rule conception (74.03%), with moral rule scores showing more homogeneous distributions. Emotion regulation scores were moderate overall (40.64%), with the most pronounced individual differences observed in the use of emotion regulation strategies, highlighting this subdimension as a potential focus for developmental support.

Pearson correlation analyses revealed significant positive associations across most subdimensions. The strongest correlation was observed between Emotion Regulation Strategies and total Moral and Social Rule Conception (*r* = 0.78, *p* < 0.001), followed by associations with Social Rule Conception (*r* = 0.74, *p* < 0.001) and Moral Rule Conception (*r* = 0.69, *p* < 0.001). Recognizing and Understanding Emotions showed moderate associations with both moral (*r* = 0.41) and social (*r* = 0.43) rule conceptions. These medium-to-large effect sizes suggest meaningful developmental linkages between emotional competence and normative awareness in this age group.

Given the exploratory nature of this study and the limitations inherent in the small sample size (*N* = 45), these associations should be interpreted with appropriate caution. The findings are best understood as preliminary, hypothesis-generating evidence that warrants replication with larger and more diverse samples. Nevertheless, the consistent pattern of strong positive correlations between emotion regulation strategies and normative awareness across all subdimensions provides a coherent and theoretically grounded basis for further investigation.

### 4.1 Limitations, implications, and future directions

This study has several limitations that should be considered when interpreting the findings. The data were collected from a relatively small and homogeneous sample of 45 children (mean age = 67.47 months) attending two public preschools. This limited and context-specific sampling framework restricts the generalizability of the results to broader socioeconomic, cultural, and geographical populations. Future research should therefore include larger and more diverse samples to explore potential moderating factors such as gender, socioeconomic background, and educational context.

Additional methodological constraints should be noted. First, the sample size (N = 45) limits the statistical power of the correlational analyses and constrains the generalisability of the observed effect sizes; replication with larger samples is essential to establish the robustness of these associations. Second, the analyses did not include potential confounders (e.g., socioeconomic status, parental education, language ability, classroom climate), so the observed associations may be partly explained by unmeasured third variables. Third, the reliance on bivariate correlations, while appropriate for the current sample size, does not allow for the examination of unique contributions of individual emotion regulation subdimensions when controlling for shared variance. Future studies with larger samples could employ multivariate approaches to disentangle these effects. Finally, multiple correlations were computed simultaneously, which may increase Type I error; future work could preregister hypotheses and apply correction procedures (e.g., Bonferroni) where appropriate.

Data collection was conducted directly by the researchers in a single assessment occasion. While this ensured procedural consistency, it may introduce rater/administrator effects and shared-method variance across measures. Future studies should incorporate independent assessors, inter-rater reliability checks, and additional data sources (e.g., teacher and parent reports, classroom observations) to strengthen measurement validity.

The cross-sectional design further limits causal inferences about the directionality between emotion regulation and moral-social rule conception. Longitudinal or experimental designs would allow for a more robust examination of how children’s emotion regulation skills evolve and interact with moral understanding over time. Integrating intervention-based approaches-such as structured emotion regulation or moral education programs-could provide valuable evidence of causal mechanisms and practical outcomes.

It should also be acknowledged that moral and social rule conception are still-developing constructs during early childhood. The age range examined (64–72 months) represents a sensitive developmental window in which these capacities are actively emerging and evolving. A single cross-sectional assessment may not fully capture the dynamic nature of these developing competencies, and children’s responses at one point in time may not reflect the trajectory of their normative understanding. Longitudinal designs that track how moral and social rule awareness develops and interacts with emotion regulation over time would provide a more complete developmental picture.

Additionally, the exclusive reliance on quantitative methods limited the depth of understanding regarding children’s emotional experiences and reasoning processes. Future research could adopt qualitative or mixed-method designs (e.g., structured observations, narrative tasks, drama-based activities) to capture a more contextualized and nuanced picture of these developmental processes.

Despite these limitations, the findings offer significant implications for early childhood education and policy. At the educational level, findings highlight the importance of integrating emotionally informed rule learning into preschool curricula. Teachers can act as emotional mediators, helping children recognize and manage emotions in moral decision-making. Structured socio-emotional learning (SEL) activities-such as role-playing moral dilemmas, storytelling, or cooperative games involving fairness and sharing-can foster both emotional regulation and moral reasoning.

For children, parents and teachers, embedding emotion-regulation programs within early intervention frameworks may help reduce behavioral difficulties and enhance social adaptation. Evidence from preventive education programs suggests that early emotion-regulation training supports later peer acceptance and school readiness (Denham et al., 2014; Graziano et al., 2007). Policymakers can build upon these insights to design comprehensive strategies that align socio-emotional and moral education goals from early childhood onward.

## Data Availability

The original contributions presented in the study are included in the article/Supplementary material, further inquiries can be directed to the corresponding author/s.
